# COVID-19 infections in day care centres in Germany: social and organisational determinants of infections in children and staff in the second and third wave of the pandemic

**DOI:** 10.1186/s12889-021-12470-5

**Published:** 2022-01-14

**Authors:** Franz Neuberger, Mariana Grgic, Svenja Diefenbacher, Florian Spensberger, Ann-Sophie Lehfeld, Udo Buchholz, Walter Haas, Bernhard Kalicki, Susanne Kuger

**Affiliations:** 1 German Youth Institute (DJI), Nockherstr. 2, Munich, 81541 Germany; 2 Catholic University of Eichstätt-Ingolstadt, Kapuzinergasse 2, Eichstätt, 85072 Germany; 3 Robert Koch-Institute (RKI), Nordufer 20, Berlin, 13353 Germany

**Keywords:** SARS-CoV-2, COVID-19, Early childhood education and care (ECEC), Germany

## Abstract

**Background:**

During the SARS-CoV-2 pandemic, German early childhood education and care (ECEC) centres organised children’s attendance in different ways, they reduced opening hours, provided emergency support for a few children, or closed completely. Further, protection and hygiene measures like fixed children-staff groups, ventilation and surface disinfection were introduced in ECEC centres. To inform or modify public health measures in ECEC, we investigate the occurrence of SARS-CoV-2 infections among children and staff in ECEC centres in light of social determinants (i.e. the socioeconomic status of the children) and recommended structural and hygiene measures. We focus on the question if the relevant factors differ between the 2nd (when no variant of concern (VOC) circulated) and the 3rd wave (when VOC B.1.1.7 (Alpha) predominated).

**Methods:**

Based on panel data from a weekly online survey of ECEC centre managers (calendar week 36/2020 to 22/2021, ongoing) including approx. 8500 centres, we estimate the number of SARS-CoV-2 infections in children and staff using random-effect-within-between (REWB) panel models for count data in the 2nd and 3rd wave.

**Results:**

ECEC centres with a high proportion of children with low socioeconomic status (SES) have a higher risk of infections in staff and children. Strict contact restrictions between groups like fixed group assignments for children and fixed staff assignments to groups prevent infections. Both effects tend to be stronger in the 3rd wave.

**Conclusion:**

ECEC centres with a large proportion of children with a low SES background and lack of using fixed child/staff cohorts experience higher COVID-19 rates. Over the long run, centres should be supported in maintaining recommended measures. Preventive measures such as the vaccination of staff should be prioritised in centres with large proportions of low SES children.

## Background

So far, Germany faced three pandemic COVID-19 waves. While the 1st wave in spring 2020 was followed by a phase of low incidence during summer 2020, the 2nd wave started approximately in CW 40/2020 and lasted until the first weeks in 2021. The 3rd wave followed on foot and was characterised by a parallel rise of the proportion of specimens diagnosed as the VOC B.1.1.7 (“British variant” resp. “Alpha”). To curb incidences, the German government ordered national lockdowns and reduced the number of children in early childhood education and care (ECEC) centres to reduce the number of contacts [1–5].

### Children’s attendance in German ECEC Centres during the COVID-19 pandemic

With beginning of the 1st wave, a “strict” lockdown was introduced during which only children with parents providing essential services (e.g. physicians or food vendors) and children in need of child welfare services (e.g. cases of maltreatment) could attend ECEC centres [5]. During the 2nd wave, a 2nd lockdown was installed from CW 51/2020 until CW 04/2021. Throughout this lockdown, all children could attend ECEC in principle. However, most federal states appealed to parents to keep their children at home if possible [1–4]. During the 3rd pandemic wave (since CW 05/2021), the closure of ECEC centres was largely dependent on the incidence of individual districts. Hence, ECEC attendance regulations differed across regional meso and micro levels.

### Transmission of SARS-CoV-2 involving young children: unclear role of preventive and hygiene measures

Before the circulation of VOC B.1.1.7 (i.e. prior to the 3rd wave), children aged 1 to 11 years old were under-represented among COVID-19 cases compared to their proportion in the general population and particularly under-represented among cases experiencing severe outcomes, such as hospitalization, requiring respiratory support or death [6]. Two systematic literature reviews conducted in 2020, i.e. before the circulation of VOC B.1.1.7, concluded that children in this age span were less susceptible than adults [7, 8]. Data on the infectiousness of children have shown equivocal results. In household studies, children were rarely identified as primary cases [6] and gave rise to a lower (secondary) attack rate ((S)AR) of 7.9% (95% confidence interval (CI), 1.7%-16.8%) compared to that of adults (15%, 95% CI, 6.2%-27%) [9]. In German ECEC centres, children with COVID-19 infections have led to an average SAR of 1.7% and small outbreak sizes (average size: 3-4 cases) [10]. An increased risk of infections in families associated with having children attending day-care centres for children aged 0 to 2 years (aOR: 1.31; 95%CI: 1.02-1.62) and kindergarten (aOR: 1.27; 95%CI: 1.09- 1.45) was found in a french case-control study [11].

Transmission of SARS-CoV-2 occurs mainly through the respiratory route. Within the respiratory route, both short and long range transmissions are believed to play a role [12–14]. Exhaled aerosols can float in the air for hours [15], and half life of viable virus in small particles is estimated as approximately one hour [16]. Once certain boundary data, such as room size, duration of exposure, number of persons exposed and type and degree of ventilation are known, it has been possible to predict the attack rate of outbreaks [17]. The role of other transmission routes, e.g. contact transmission, remains controversial. Ferretti estimates that 10% of transmissions may be due to “environmental” factors, i.e. contact transmission [18]. Meyerowitz concludes that there is currently no conclusive evidence for fomite or direct contact transmission of SARS-CoV-2 in humans [14].

Conversely, at least within households, ventilation was shown to prevent secondary infections [19]. These findings have led to the recommendations to keep a minimum distance of 1.5 meters to other persons, wear masks and ventilate rooms where several persons are present at the same time. In principle, these recommended behaviours provide protection in the context of ECEC centres as well. However, as most transmission studies have been conducted with adults, the evidence base for preventive recommendations with children is largely unexplored. For preschool children, it cannot be expected to keep a distance of 1.5 meters to peers or staff, nor to wear masks. Recommended measures for ECEC centres have thus focused on organisational changes, e.g. the change of group concepts as well as hygiene recommendations for staff and parents.

Before the pandemic, the following (pedagogical) group concepts typically existed in German ECEC settings: (i) fixed groups, (ii) open concept (children can freely choose and switch between rooms and peers), and (iii) partly open concept, e.g. a fixed group in the morning and free roaming in the afternoon. These concepts leave all options open how staff is assigned to groups. An important organisational change in ECEC centres was the recommendation to switch not only to fixed groups, but also to keep pedagogical staff of a given group constant (fixed staff assignment to a particular group). In addition, infection control and hygiene recommendations included regular ventilation of rooms and regular disinfection of surfaces [20].

### Research questions

We put a focus on understanding (1) relevant determinants, risk and protective factors for COVID-19 occurrence in ECEC centres, (2) whether factors associated with infections differ between children and staff, and (3) whether the relevant factors differ between the 2nd and 3rd wave, as the 3rd wave was largely driven by the mutated VOC B.1.1.7 [21] with possibly different epidemiological properties.

## Methods

### Study design

The German Youth Institute (“Deutsches Jugendinstitut”; DJI) and the Robert Koch-Institute (the national public health institute; RKI) joined forces to monitor the situation of preschool children during the pandemic in the so called “Corona-KiTa-Study” (ECEC centre registry). In one of the project modules, the DJI established a novel assessment system drawing information directly from ECEC centres (the so-called “KiTa-Register” which translates into “ECEC centre registry”). The ECEC centre registry has been set up to monitor the situation of ECEC centres during the pandemic, starting in CW 36, in August 2020. From CW 36 in August 2020, managers of all ECEC centres in Germany were asked to fill out a weekly questionnaire. Data could be provided for the current week and, in case centre managers had missed the last survey, also for the week before. Data collection is still ongoing. For this contribution, the period of analysis ends in CW 22 (June 2021) with the end of the 3rd wave. In consequence, the longitudinal data of the study reported here comprises 37 timepoints. This allows comparing the 2nd wave (from CW 36/2020 to CW 04/2021) with the 3rd wave (from CW 05/2021 to CW 22/2021). The online surveys were answered by centre managers with regard to the whole ECEC centre. Managers of all ECEC centres in Germany were eligible for participation. The ECEC centre registry has been set up to monitor the re-opening and closing of ECEC centres and the current share of children and staff attending in reference to the attendance rate before the pandemic on a weekly basis. Furthermore, information on COVID-19 infections in ECEC centres and the implementation of infection control and hygiene measures has been collected.

### Data

To investigate possibly predictive and protective factors of COVID-19 infections in ECEC centres, this paper uses the panel data set of ECEC centres in Germany within the ECEC centre registry. The survey comprises a baseline questionnaire handed out to the ECEC centres at the timepoint of registration. The baseline questionnaire asks leaders about registration to collect basic time-constant information such as the type of provider, the centre’s proportion of children from households with low socioeconomic status (SES) and the group concept prior to the pandemic. The subsequent weekly questionnaire collects information about the current week and contains time-varying variables such as the number and age of children currently attending the ECEC centre, the number of staff working at the ECEC centre in general as well as in the current week, the currently applied group concept, if staff was assigned strictly to groups (fixed staff assignment, only asked if ECEC currently use a fixed or partly open group concept), application of certain hygiene measures, as well as the number of children, staff and parents who were tested positive for COVID-19. Until CW 22/2021, 8,500 of roughly 54,000 ECEC centre managers participated at least once with an average number of 29.5 reported weeks.

### Dependent variables

We use the number of reported infections per week as a dependent variable. To measure infections in staff and children, ECEC centre managers were asked if they had any new laboratory confirmed cases of COVID-19 in children or staff. Infections were reported for staff members and for children separately. For data protection reasons, detailed information on infections in staff was only asked in ECEC centres with at least 7 staff members (which applies to 97% of our sample). The serial interval of COVID-19 (i.e. the average interval from the onset of illness in an infectious / case to the onset of illness of a case infected by that case) is estimated to have a duration of 5 days [22, 23]. After laboratory diagnosis, 1-3 days may pass until the result of a test is available at the district health department [24]. Hence, we included the variable “number of infections” with a lead of one week in our models and estimated the rate of infection in CW X+1 with data from CW X. We do not analyse infections in parents, as the link to the ECEC centre is not necessarily given here.

### Independent variables

As possible predictors for the number of reported infections, we use variables which are either time-constant or time-variant.

#### Time-constant variables

*Type of provider:* The type of provider (public, private for-profit, ecclesiastical or other non-profit) of the ECEC centres is included, since it might be systematically associated with the implementation of particular hygiene measures.

*Socioeconomic status:* COVID-19 infections are known to follow a social gradient [25, 26]. To control for social composition, ECEC managers were asked to estimate the proportion of children with low socioeconomic status (SES) on a 4-point Likert scale (i.e. below 10% children with low SES background, 11% to 30%, 31% to 60%, or above 60%, respectively).

*Group concept prior to the pandemic:* To grasp differences in the set-up of the institutions which might make it difficult to implement certain measures such as e.g. group separation, we include the type of pedagogical group concept before the pandemic in our model.

We include these variables as time-constant dummy variables in the models.

#### Time-varying variables

*Currently applied group concept:* Managers indicated which group concept was currently in use. We distinguished between the three categories “open group concept”, i.e. children can mix freely, “partly open”, i.e. children are assigned to groups in the morning, but can mix in the afternoon, and “fixed”, i.e. strict assignment of children to only one group.

*Infection control and hygiene measures:* Managers specified which other measures they implemented in their centre in the current week: (1) Regular ventilation of the rooms, (2) regular surface disinfection (e.g., furniture surfaces, door handles or toys), and (3) a fixed staff assignment to groups (only asked for fixed or partly open group concepts). For each measure, managers indicated whether it was regularly applied or not in this week with the help of a yes/no-question.

#### Control variables

*7-day incidence at district level:* Laboratory confirmed COVID-19 cases are notified to the local health authority (LHA) in accordance with the German Protection against Infection Act (“Infektionsschutzgesetz”, IfSG)^1^. The LHA transmits reported cases from all 401 German districts via the respective federal state health authority to the RKI. The 7-day incidence includes the number of newly reported cases within seven days in a population of 100,000.

*Number of Children:* Managers indicated per week how many children aged 0-2, 3-6, and 7 years and older attended the ECEC centres. These numbers may change due to (perhaps only regionally observed) holidays as well as regional outbreaks and measures taken by the federal states or districts.

We use the latter two variables, 7-day incidence on district level and number of children attending, to control for risk of exposure to the virus.

### Statistical analysis

Our data provide various information about ECEC centres, namely *time-constant* variables related to the centre (from the baseline questionnaire), average differences in the time-varying variables *between* ECEC centres and changes *within* a centre. Since the Poisson distribution is known to approximate incidence counts from a wide variety of underlying processes [27], we use a random-effect panel poisson model for count data with demeaned data to approximate incidence counts (see formula 1). That allows us to estimate the effects of time-constant variables, between-unit differences and within-unit changes on the incidence count at the same time [28, 29].

1 $$ {}\begin{aligned} log(E(y_{i,t+1})) &= \alpha + \beta_{1within}(x_{it}-\bar{x}_{i})+\beta_{2between} \bar{x}_{i}\\ &\quad+\beta_{3}z_{i}+\upsilon_{i1}+\upsilon_{1t} \end{aligned}  $$

We specify our model with a leading y variable (t+1). *β*_1_ estimates within-units effects, hence what happens in the next weeks after a unit e.g. decides to change its group concept from fixed to open. *β*_2_ estimates between-units effects, hence the effect of e.g. having an open group concept over all the weeks under study. *β*_3_ estimates effects of time-constant variables, e.g. of having applied an open group concept before the pandemic. *υ*_*i*1_ and *υ*_1*t*_ are unit- and time-fixed effects. Exponential coefficients could be interpreted as incidence rate ratios, hence how much the expected count changes multiplicatively when x increases by one.

As the occurrence of COVID-19 infections varies by time and region, we include a district’s weekly 7-day incidence as (log) exposure A [30] with a regression coefficient constrained to 1, allowing the model to represent rates instead of counts. This is equivalent to standardizing the dependent variable with the offset variable (see formula 2, equivalent with formula 3).

2 $$ {}\begin{aligned} log(E(y_{i,t+1})) &= \alpha + 1*log(A)+\beta_{1within}(x_{it}-\bar{x}_{i})\\&\quad+\beta_{2between}\bar{x}_{i} +\beta_{3}z_{i}+\upsilon_{i1}+\upsilon_{1t} \end{aligned}  $$

3 $$ {} \begin{aligned} log\left(\frac{E(y_{i,t+1})}{{A}}\right) &= \alpha + \beta_{1within}(x_{it}-\bar{x}_{i})+\beta_{2between}\bar{x}_{i}\\&\quad+\beta_{3}z_{i}+\upsilon_{i1}+\upsilon_{1t} \end{aligned}  $$

By doing so, we include the assumption that an ECEC centre in a district with twice as many infections also reports twice as many cases in its ECEC centres. Since the likelihood of an occurrence of a COVID-19 infection does not only depend on regional conditions, but is also strongly dependent on the number of persons in the respective facilities, we add within- and between-effects for the number of children of all age groups (0-2, 3-6, 7 plus) in our model. As we tend to refrain from interpreting these variables directly, they are included in the model as mere controls and are only shown in the Supplementary Material.

## Results

### COVID-19 infections in ECEC centres over time

**Fig. 1:**
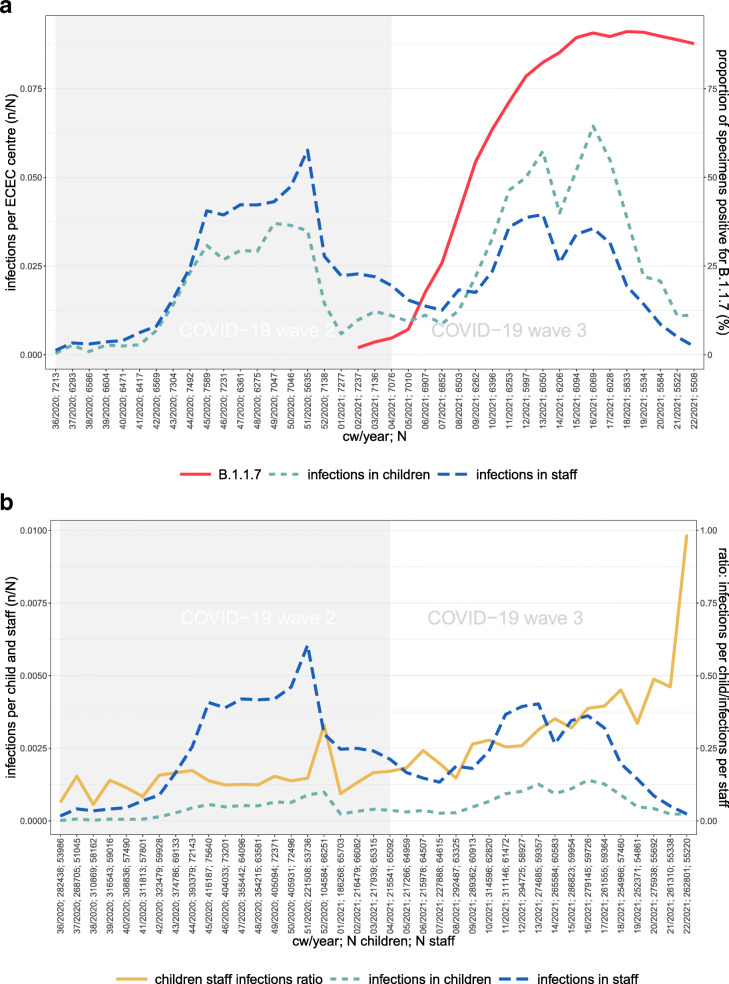
Infections in children and staff. **a** Number of infections (n) in children and staff in ECEC centres (N) per week, (n/N per week) and proportion of specimens in German laboratories positive for B.1.1.7 (%) **b** Number of infections (n) in all children/staff (N) currently in the ECEC centre per week. Source: Survey data (ECEC centre registry) collected by the DJI, own calculations. RKI Data on B.1.1.7; Robert Koch-Institut (2021): Bericht zu Virusvarianten von SARS-CoV-2 in Deutschland (April). Second wave assumed to last from calendar week (CW) 36/2020-04/2021, and third wave from CW 05/2021-22/2021

Figure 1a shows the development of COVID-19 infections in children and staff between CW 36/2020 and CW 22/2021. The grey area in Fig. 1 demarcates the 2nd wave (including CW 04 in 2021), the remaining white area (CW 05-22 in 2021) demarcates the 3rd wave. Our wave definitions do not exclude weeks with low incidence rates as we want to measure within and between effects, hence we need to include the lead time before incidences rise.

In the 2nd wave of the pandemic, the weekly number of infections in staff per ECEC centre exceeds the number of infections in children per ECEC centre. From CW 09/2021, the two lines cross and the number of infected children per ECEC centre surpasses that of staff. To account for the number of children and staff attending week by week, Fig. 1b shows the proportion of infections in ECEC staff and children, respectively, as well as the ratio of the proportion of infections among children relative to the one in staff, the latter could be interpreted as a rate ratio. Overall, the rate of infections in staff is higher compared to that of children, but the ratio clearly increases in the 3rd wave, and further rises when infections go back in the last weeks of wave 3.

### Attendance, protection and hygiene measures in ECEC centres over time

In the following, we briefly discuss the development of our time-varying indicators. As we specify our model with a 1 week lead, explanatory variables are only considered up to CW 21/2021. Respective figures and tables are included in the Supplementary Material. Regarding *children’s attendance*, prior to the 2nd wave, the average ECEC centre each week cared for roughly 12 children aged 0 to 2 years, for 45 children aged 3 to 6 years and for 3-4 children aged 7 years or older (see Supplementary Figures S1a, S1b and Tables S2, S3 for details). The latter number might seem low, but our data collection excludes all ECEC centres with after-school childcare that do not provide ECEC services for younger children. Overall, we found only little variation in the number of children attending child care in the weeks 36 to 50 in 2020. We observe a steep drop in children’s attendance in CW 50 to 52 in 2020, as the German federal government ordered a nationwide lock-down in which most federal states appealed to parents to keep their children at home if possible [1–4]. Roughly half of the children returned after Christmas, while the 2nd half returned in CW 8/2021, followed by two minor increases in CW 15 and 20/2021.

The share of ECEC centres applying a fixed group concept (see Supplementary Figures S2a, S2b and Tables S2, S3) increased from 62% in CW 36/2020 to 80% in CW 21/2021. At the same time ECEC centres with a partly or fully open concept decreased from 29% to 15% and 8% to 5%, respectively.

Considering *infection control and hygiene measures*, regular ventilation was conducted in most ECEC settings during the whole period and decreased only slightly from 100% of institutions in CW 36/2020 to 97% in CW 21/2021 (see Supplementary Figures S3a, S3b and Table S2, S3 in the Supplementary Material). The vast majority of ECEC centres indicated to have disinfected surfaces regularly during the whole time under study, with only a small drop from 92% in CW 36/2020 to 88% in CW 21/2021. Only 63% of ECEC centre managers reported that their staff was assigned strictly to a fixed or partly open group in CW 36/2020, but this share increased to 69% in CW 21/2021. Despite these relatively small changes over time, we found considerable within-variance in the latter variable. We observed simultaneous changes in different directions, as some ECEC centres started and others stopped infection control and hygiene measures in the same week, leading to a low change in overall percentages.

### Factors associated with COVID-19 infections in ECEC centres

**Fig. 2:**
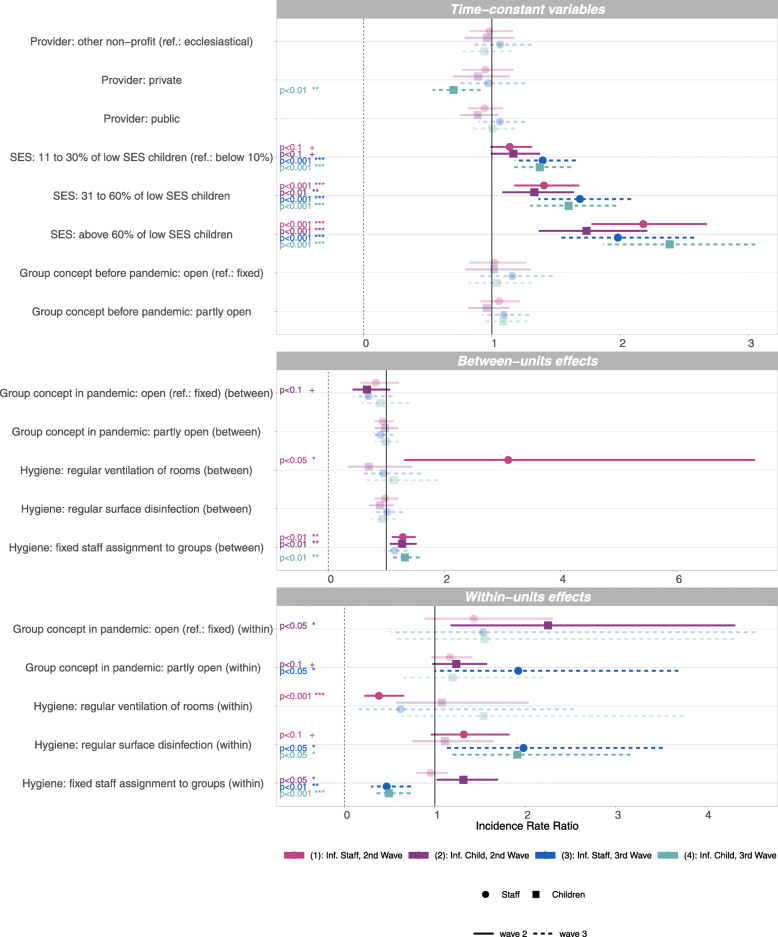
Incidence rate ratios of predictors for infections (Inf.) in staff and children in 2nd (Model 1,2) and 3rd wave (Model 3,4) of the pandemic. ^∗∗∗^*p*<0.001; ^∗∗^*p*<0.01; ^∗^*p*<0.05; ^*†*^*p*<0.1. Source: Survey data collected by the DJI (ECEC centre registry). Second wave assumed to last from calendar week (CW) 36/2020-04/2021, and third wave from CW 05/2021-22/2021. REWB poisson model with two-way fixed effects, offset for district 7-day incidence (data collected by the Robert Koch-Institute), dependent variable with 1 week lead. Coefficients are displayed as incidence rate ratio, confidence bounds (95%) as bars, effects that do not reach the threshold of *p*<0.1 are transparent, significant effects are shown as opaque. Controlled for number of children in different age groups (within and between effects), see Supplementary Table S1 for full model, own calculations

Figure 2 shows the results from the REWB models. It contains coefficients from models analysing the number of infections within different time frames (i.e. 2nd and 3rd wave of the pandemic) in both staff and children, hence four coefficients per explaining variable. The coefficients are shown as incidence rate ratios. The point estimates are marked with a dot (for infections in staff, Model 1 and 3) or a square (for infections in children, Model 2 and 4). Horizontal lines indicate the confidence bounds (95%), where the linetype marks the wave, i.e. 2nd wave (solid line, Model 1 and 2) and 3rd wave (dashed line, Models 3 and 4). Models 1 and 2 cover a 21-week time frame from CW 36/2020 to CW 04/2021 (i.e. 2nd wave, the grey area in Fig. 1), while Models 3 and 4 cover a 17-week time frame from CW 05/2021 to CW 22/2021 (i.e. 3rd wave, the white area in Fig. 1). Significant coefficients (^*†*^*p*<0.1) are printed in opague, non significant coefficients are printed in transparent colours. Significance levels are further printed as text in the corresponding colours on the left side of Fig. 2.

#### Results for time-constant variables

We do not find significant differences between *types of providers*, except a negative effect of private providers on infections in children in wave 3 (Model 4). The *proportion of low SES children* is found to be a significant predictor for the rate of infections in staff as well as in children in all four models for all three categories of higher SES (compared to ECEC centres with under 10% SES children). The higher the proportions of children with a low SES background, the higher the infection rate. For the category 11% to 30% of low SES children (compared to less than 10% low SES children), effects are only significant at the 10% significance level (^*†*^*p*<0.1) in Models 1 and 2 (i.e. 2nd wave). Effect sizes of SES tend to be larger in wave 3 compared to wave 2 for both infections in staff and children, with one exception: for infections in staff, the effect of the largest proportion of children with low SES background is larger in wave 2 than in wave 3. The variable *group concept prior to the pandemic* did not yield any significant effects.

#### Results for time-varying variables: between-units effects

Between-units effects compare averages across CWs between ECEC centres and are prone to endogeneity, that is, a positive effect (indicating more infections) of implementing a protection measure could stem from the fact that centres in counties with very low COVID-19 incidences tend to implement protection measures to a lower extent than centres in counties with higher incidences.

We do not find significant between-units effects regarding the *currently applied group concept*, with the exception of a negative effect of open group concept (compared to fixed groups) at the 10% level for infections in children in the 2nd wave (Model 2), indicating that, controlling for the factors in the model, ECEC centres with low infection rates tend to apply open group concepts more often.

We find a significant positive between-units effect for *regular ventilation* for infections in staff in wave 2 (Model 1), but no between-unit effects of regular surface disinfection. We further find positive between-units effects for *fixed group assignment of staff* (Models 1, 2, and 4, i.e. wave 2 for staff and children and wave 3 for children). The positive effects indicate that, controlling for the factors in the model, ECEC centres that report to adhere to the recommendation to ventilate rooms more frequently or have on average more often a fixed staff assignment to groups (i.e. they implement these measures in more weeks) also report higher average infection rates in the respective settings.

#### Results for time-varying variables: within-units effects

Next, we describe time-varying coefficients for within-units effects which estimate the effect of a within-unit change. For example, in the case of the variable *group concepts*, we estimate what happens if ECEC centres switch from a fixed to an open group concept between weeks. Results thus imply a temporal association of events within an ECEC centre.

Considering the *group concept*, we find significant within-units effects. Switching to an open concept (from a fixed concept) is associated with a significant increase in the number of infections among children in the 2nd wave (Model 2). Switching to a partly open concept (from a fixed concept) is associated with a significant increase of infections in children in the 2nd wave (Model 2), and more so, with increasing infections in staff in the 3rd wave (Model 3).

Starting the implementation of *regular room ventilation* is significantly associated with fewer infections in staff in wave two (Model 1), but has no significant effects on the infection rates in any other model (Models 2, 3 and 4).

Implementing *regular disinfection of surfaces* from one week to the next is associated with a significant increase of infections among staff and children in the 3rd wave (Model 3 and 4), and less so, with increasing infections among staff in the 2nd wave (Model 1, 10% level). Further analysis showed that this effect is likely to stem from ECEC centres which started disinfection when local district incidences were starting to rise rapidly. Centres that reported the implementation of surface disinfection have a lower district average COVID-19 incidence before they start the measure, and a higher district average after having started with disinfection. Hence, we assume that the start of surface disinfection was a reaction to locally rising incidence rates, which, in turn, where followed by higher infection rates in ECEC centers.

Implementing *fixed group assignment of staff* is associated with significantly fewer infections in both staff and children in wave 3 (Model 3 and 4). In the 2nd wave models, we found no effect for infections in staff, but a significant positive effect for infections in children (Model 2). The small positive effect in Model 2 might again be read as anticipation, hence ECEC centres start with fixed staff assignments to groups in the face of locally rising incidence rates, presumably reacting to regional orders for implementation which are linked to rising incidence rates. Considering protective measures, the very low number of within changes, especially in ventilation and disinfection, leads to a strong dependence on few observations only and the effects should therefore be interpreted with caution.

To challenge our results, we additionally ran a variety of models as robustness checks, i.e. several models with protective measures only (Supplementary Tables S5-S8), models that include the offset as Covariate (Supplementary Table S9), models with alternative wave cut-off points (Supplementary Tables S10 and S11) as well as models that include further individual and regional controls, i.e. the number of infections in parents in the corresponding ECEC centre as well as regional population density and regional median income (Supplementary Table S12). All tests by and large confirm the above results in relation to the sign and the effect strength and can be found in the Supplementary Tables S5-S12.

We further tested if our data fits the poisson specification by analyzing the conditional mean and variance of the outcome variables in our models, finding underdispersion in all our models. Underdispersion leads to overestimated standard errors in poisson models [31]. As we prefer to not correct for that bias, e.g. by using a quasipoisson model (which would lead to smaller confidence bounds), our significance levels could be characterised as conservative. This approach seems appropriate, especially in view of the fact that our data does not represent a true random selection. We tried alternative specifications for underdispersed data, i.e. using a generalised poisson distribution (see Supplementary Table S13) that by and large confirmed our results. Notable exceptions are the within-effect of fixed staff assignment for infections in children in wave 3 (Model 4) which is smaller and insignificant as well as the within effects of surface disinfection, which are insignficant when a generalised poisson distribution is assumed.

## Discussion

Our study investigated factors associated with infections in staff and children in ECEC centres in Germany during the 2nd and 3rd wave of pandemic. We found ECEC centres with a larger proportion of children with a low SES background to have the highest risk of infections, and we found this effect to be increasing in the 3rd wave compared to the 2nd wave. That the socio-economic status plays a role for both incidence and adverse outcome of COVID-19 has been shown in various studies [32–34]. In Germany, the effect only became apparent after the first wave [26]. However, most of these studies are ecological by design. Although also in our study the association of infections with socio-economic status is not shown on an individual level, the degree of evidence is high as we were able to collect very detailed information on individual ECEC centres.

Changing the *group concept* towards a more restrictive group separation was one of the essential recommended or ordered measures since the beginning of the pandemic. A large proportion of ECEC centres in our sample followed this recommendation, especially in the 3rd wave (Supplementary Figure S2). The significant associations of switching from a fixed to an open or partly open concept shows that failure to follow these recommendations puts childrens’ and staffs’ health at risk.

It is well recognised that the main route of SARS-CoV-2 transmission is through the respiratory mode, and that airborne transmission is of major importance [12–14]. Our findings tend to confirm this for ECEC settings. In our data, regular ventilation had a significant protective effect on infections in one of the four models, specifically on staff in the 2nd wave (model 1). Overall, the implementation of *regular ventilation* as a protective measure in ECEC centres was quite ubiquitous with only very little variance. Hence, insignificance is probably due to very low case numbers resulting in low overall variance. Nevertheless, even if regular ventilation was widely implemented during the whole period under study, we observed a small, but constant decline in implementation.

Taking into account the strong evidence from case, cluster and outbreak reports which indicate that proximity and ventilation are key determinants of transmission risk [14], contact or fomite transmission can be regarded as an unusual mode of transmission. In our study, we even find significant positive within results for regular surface disinfection in our models, i.e. more surface disinfection is associated with more infections. Hence our study provides no evidence that surface disinfection prevents COVID-19 cases, but on a theoretical level and based on the existing evidence, it is unlikely that it creates cases. We believe that the most likely explanation for these results is that ECEC managers anticipate or quickly react to local outbreaks or increasing COVID-19 incidences by implementing hygiene measures, such as the disinfection of surfaces. Hence, we do not know to which extent our results are biased due to anticipation.

In analogy to the measures recommended for elderly and health care, fixed group allocation has been recommended also for schools and ECEC settings [35]. The main reason is to limit the number of effective close contacts to reduce the probability of infection. When some ECEC centres allowed more contacts by switching from a fixed to a more flexible staff assignment or from a fixed to a partly open group concept, e.g. in CW 07 and 08 in the 3rd wave (see Supplementary Figure S3b, S2b), this was associated with an increase of infections. Thus, our results support recommendations to maintain fixed staff assignment and fixed groups in ECEC centres whereever possible as long as the pandemic situation continues.

Although it is unknown which infections in the data are due to a VOC, the 3rd wave was dominated increasingly by the VOC B.1.1.7, which is associated with increased transmissibility [36]. This again geos along with our finding that the effects of SES are stronger in wave 3, and also with our finding of stronger effects of fixed staff assignment in the 3rd wave. As the VOC B.1.1.7 started to dominate, it became particularly important to keep up contact restrictions among staff, between child groups, and between staff and parents.

## Limitations

We acknowledge as a general limitation that a manager’s decision to implement specific measures in their ECEC centre (and the according report in our questionnaires) is not always followed and translated into every-day practice by all staff members. The responding managers also did not receive precise instructions as to when exactly a hygiene measure is considered as implemented (e.g. information on frequency of a certain measure). However, detailed hygiene plans with descriptions of the procedures were handed out by the health authorities, at least for the separation of groups and the assignment of staff. In the questionnaire, we only asked if a measure was regularly applied or not in this week with the help of a yes/no-question. Further, recall bias is a widespread topic in COVID-19 studies [37], as a lot of surveys include retrospective questions on pre-pandemic conditions. In our study, only the questions regarding group concept before the pandemic and regarding SES of the children refer to pre-pandemic conditions, and both questions aim to assess structural characteristics of the ECEC centre rather than characteristics that are more subjective, like e.g. staff’s beliefs. Our main variables are, in turn, asked on a weekly basis where recall bias should only play a minor role. Nevertheless, for the above reasons, our weekly data on hygiene measures are subject to a certain degree of uncertainty.

Further, it could be argued that some measures are conceptually similar, e.g. a fixed group concept (with strict separation of children) and a fixed staff assignment which includes that also staff does not move between groups. As both show significant effects, this suggests that strict contact restrictions are likely to be one of the most effective protective measures, especially in the 3rd wave. It must be further mentioned that there is a certain chance that especially the results of surface disinfection and ventilation are driven by very few units within-changes (Supplementary Figure S3b), as is further shown in the robustness section in the Supplementary Material (e.g., the within effect of surface disinfection is dependent on the wave definition, see Supplementary Tables S10 and S11). This limitation does not hold true for the effect of fixed staff assignment on infections in staff, which remains significant in wave 3 in all robustness checks. We therefore strongly support the recommendation to keep up fixed staff assignment in all ECEC centres wherever possible as long as the pandemic situation continues.

## Conclusions

The result that about one third of the ECEC centres in the ECEC centre registry have not implemented the recommended fixed staff assignment is likely associated with well-known structural problems of many ECEC centres in Germany. ECEC centres have to deal with limited staffing overall and within groups and a shortage of professional staff recruitable on the labour market for years [38]. Because of this, the measure was only recommended but not prescribed by law. In order to better prepare ECEC centres for such exceptional situations in the future, it is essential to finally eliminate the staff shortage that was already prevalent before the pandemic.

In summary, our results suggest that the COVID-19 pandemic affects ECEC centres particularly when they were attended by children with low SES. The social gradient of COVID-19 does not stop at the ECEC centre’s door, indicating that children, families and staff in corresponding centres need special support. Although many ECEC centres are struggling with staffing difficulties it is important to maintain – to the best possible degree – fixed group assignments among children and fixed staff assignments to groups. In addition, generous and frequent ventilation may aid in preventing infections. As vaccinations become increasingly available and booster vaccinations will probably become necessary in the light of further SARS-CoV-2 variants, particularly staff of ECEC centres with a large proportion of children with a low SES background should be prioritised.

## Data Availability

Anonymized data will be passed on to external researchers after the project has been finalized (presumably June 2022), provided that they are used for the purpose of scientific secondary and subsequent use and under the conditions of the DJI Research Data Centre (incl. deletion periods, thematic limitation, citation, etc.). The anonymised survey data and research results are stored for 10 years as proof of good scientific practice and in accordance with the funding regulations.

## Abbreviations

CW: Calendar week
DJI: German Youth Institute (Deutsches Jugendinstitut)
ECEC: Early childhood education and care
IfSG: German Protection against Infection Act (Infektionsschutzgesetz)
LHA: Local health authority
REWB: Random-effect-within-between
RKI: Robert Koch-Institute
SES: Socioeconomic status
VOC: Variant of concern

## Footnotes

1 https://www.gesetze-im-internet.de/ifsg/__6.html, visited 13.4.2021
